# Beyond Vision and Hearing: A Case Report of Wolfram Syndrome

**DOI:** 10.7759/cureus.65107

**Published:** 2024-07-22

**Authors:** Jeyapriya U, Jennie Santhanam, Ramachandran RM, T Saideekshit, Meenakshi Sundari SN

**Affiliations:** 1 Internal Medicine, Sri Ramaswamy Memorial (SRM) Medical College Hospital and Research Center, SRM Institute of Science and Technology (SRMIST), Chengalpattu, IND; 2 Internal Medicine, Karpaga Vinayaga Institute of Medical Sciences and Research Center, Chengalpattu, IND

**Keywords:** wfs 1 gene, wolfram syndrome, sensorineural hearing loss, optic nerve atrophy, juvenile diabetes mellitus

## Abstract

Wolfram syndrome (WFS) is an uncommon autosomal recessive neurodegenerative disorder characterized by diabetes mellitus, diabetes insipidus, optic nerve degeneration, hearing impairment, and other abnormalities. Additionally, a portion of individuals experience neurological, endocrine, behavioral, and urinary tract disorders that make management more challenging. Here, we present a 22-year-old male who was diagnosed with type 1 diabetes at the age of 4 and received treatment with basal-bolus insulin therapy. He had blurring of vision and hearing loss at 13 years of age, and our evaluation revealed optic atrophy and sensorineural hearing loss. He had polydipsia and polyuria (intake/output of 5-6 L/day) despite a fairly controlled blood glucose level. Serum anti-diuretic hormone (ADH) was done, which confirmed the diagnosis of central diabetes insipidus. His sonogram and urinary flow studies revealed bilateral hydroureteronephrosis with reflux uropathy. We diagnosed him with neurogenic bladder disorder with detrusor sphincter dyssynergia. This patient had an early onset urological disorder with involvement of eyes and ears, with diabetes mellitus and diabetes insipidus, which satisfied the criteria of WFS. The genetic test confirmed the diagnosis. He is currently being managed with insulin and desmopressin.

## Introduction

Wolfram syndrome (WFS), an uncommon genetic disorder inherited in an autosomal recessive manner, is frequently known by the acronym DIDMOAD (diabetes insipidus, diabetes mellitus, optic nerve atrophy, and deafness). It is marked by progressive neurologic deterioration, juvenile-onset diabetes, and endocrine dysfunction [1]. To diagnose DIDMOAD syndrome, a patient must fulfill either two major criteria or one major and two minor criteria. The two major criteria include the onset of diabetes mellitus and the occurrence of optic nerve degeneration before the age of 16. The minor criteria encompass a loss-of-function mutation in the WFS-1/CDGSH iron-sulfur domain-containing protein 2 (CISD2) gene, sensorineural hearing loss, neurological symptoms like ataxia, epilepsy, cognitive impairment, neuropathy, diabetes insipidus, diabetes mellitus after the age of 16, optic atrophy after the age of 16, and a family history of WFS [2]. This typical case meets the clinical criteria for diagnosing WFS, and genetic testing confirms the diagnosis. Among the symptoms of the syndrome, neurogenic bladder and abnormalities in urodynamic function require careful consideration. Dysfunctions of the urinary tract may result in end-stage renal disease with fatal consequences. The significance of early detection and treatment of background urological manifestations in such patients is highlighted by this case report.

## Case presentation

**Figure 1:**
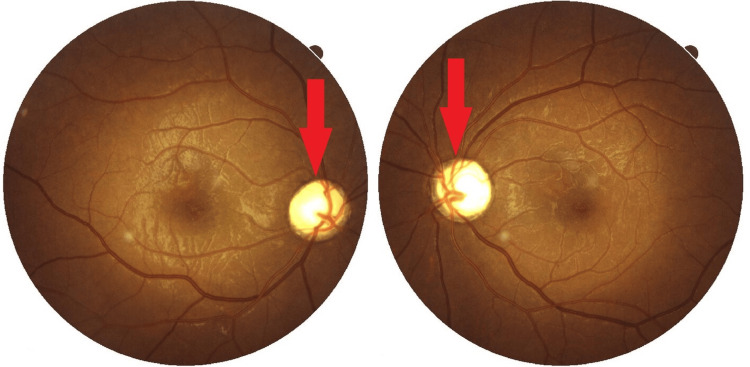
Fundus of both eyes showing bilateral optic atrophy (two red arrows).

This case study involves a 22-year-old man who was born of a second-degree consanguineous marriage. He has a younger sister without any abnormalities. He has been receiving insulin therapy for type 1 diabetes mellitus since he was four years old. Seven years before presentation to our hospital, he experienced progressive vision difficulties that left him severely visually impaired. His vision was not evaluated because he was from a remote village. He also reported an increased frequency of painless micturition over the preceding six months. At the time of admission, he was severely dehydrated, and his urinary output was 8 L/day with low urine osmolality. Ophthalmological evaluation using a pinhole revealed that visual acuity in his right eye with correction was 2/60, whereas in his left eye, it was 3/60. In an Ishihara chart color vision test, both eyes had a score of 0/25, indicating his color vision was severely impaired. Upon fundus examination, no signs of diabetic retinopathy were present, but he had a pale optic disc in both eyes indicating bilateral optic atrophy (Figure 1).

An audiology examination revealed sensorineural hearing loss on both sides. A gait examination of the patient revealed minor ataxia and difficulty in tandem walking.

**Figure 2:**
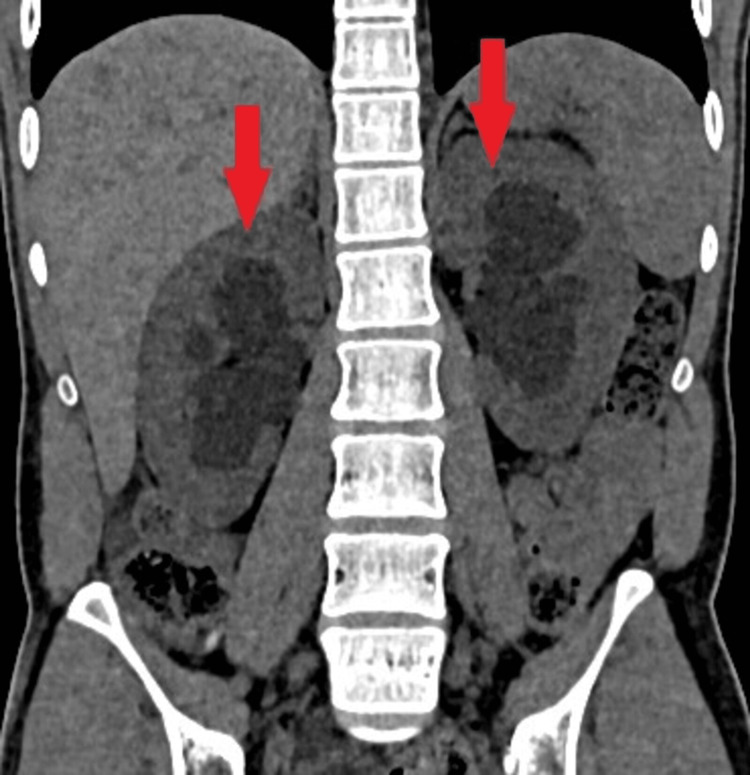
CT scan of the abdomen in a coronal section showing bilateral hydroureteronephrosis (two red arrows). CT: Computed tomography

A suspicion of WFS was raised in light of the primary criteria of bilateral optic atrophy and juvenile diabetes. With a random blood sugar reading of 560 mg/dL, the patient initially showed signs of hyperglycemia. We attributed the polyuria to osmotic diuresis due to hyperglycemia. Polyuria continued even after achieving a euglycemic state with insulin. A computed tomography (CT) scan of the abdomen showed both kidneys were bulky, with signs of bilateral hydroureteronephrosis (Figure 2). 

**Figure 3:**
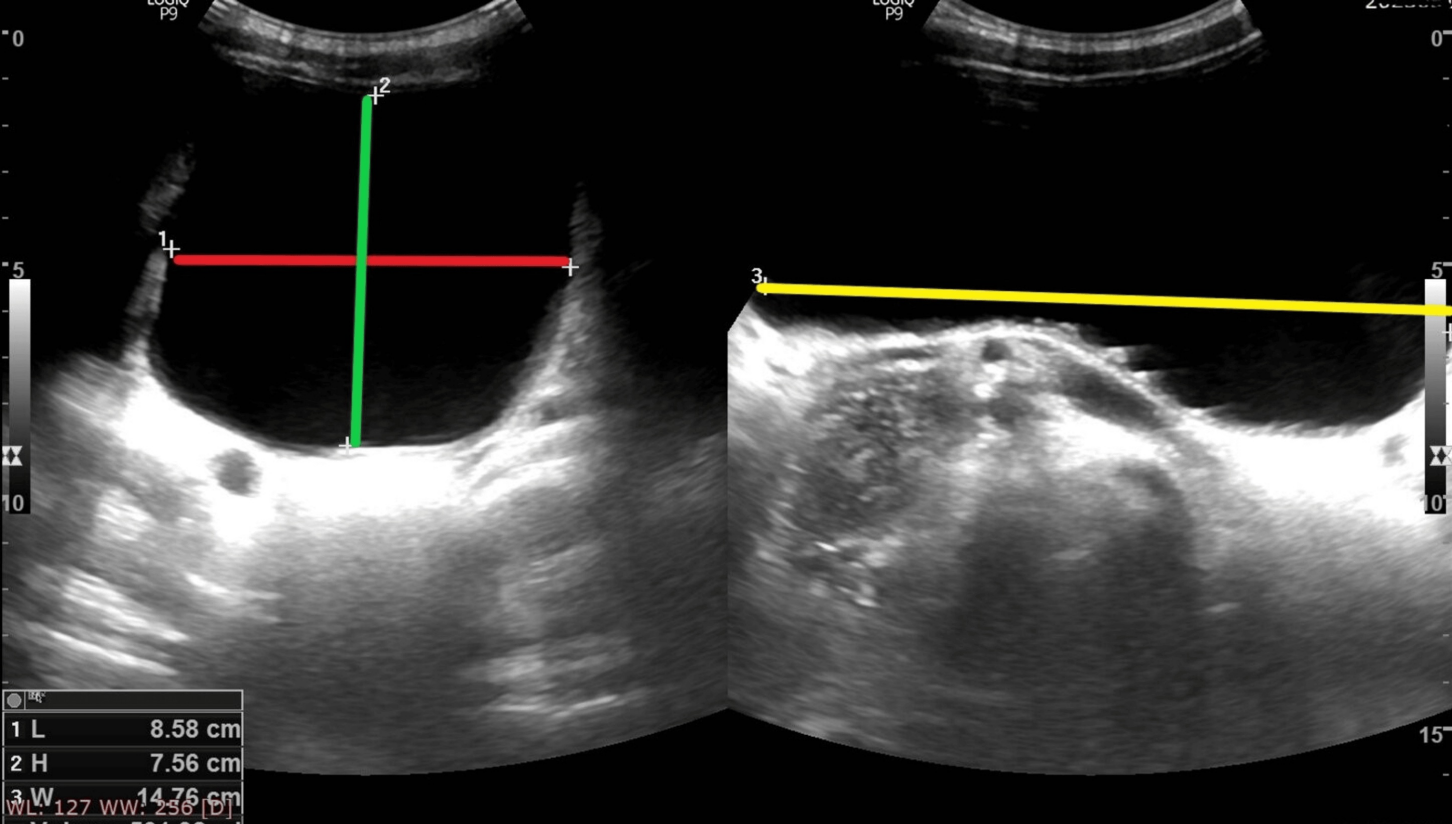
Ultrasonography of the pelvis showing significant post-void residual urine volume. The red, green, and yellow lines respectively indicate the length, height, and width of the urinary bladder.

Ultrasonography (USG) of the pelvis showed a significant post-void residual volume of 501 mL (Figure 3).

**Figure 4:**
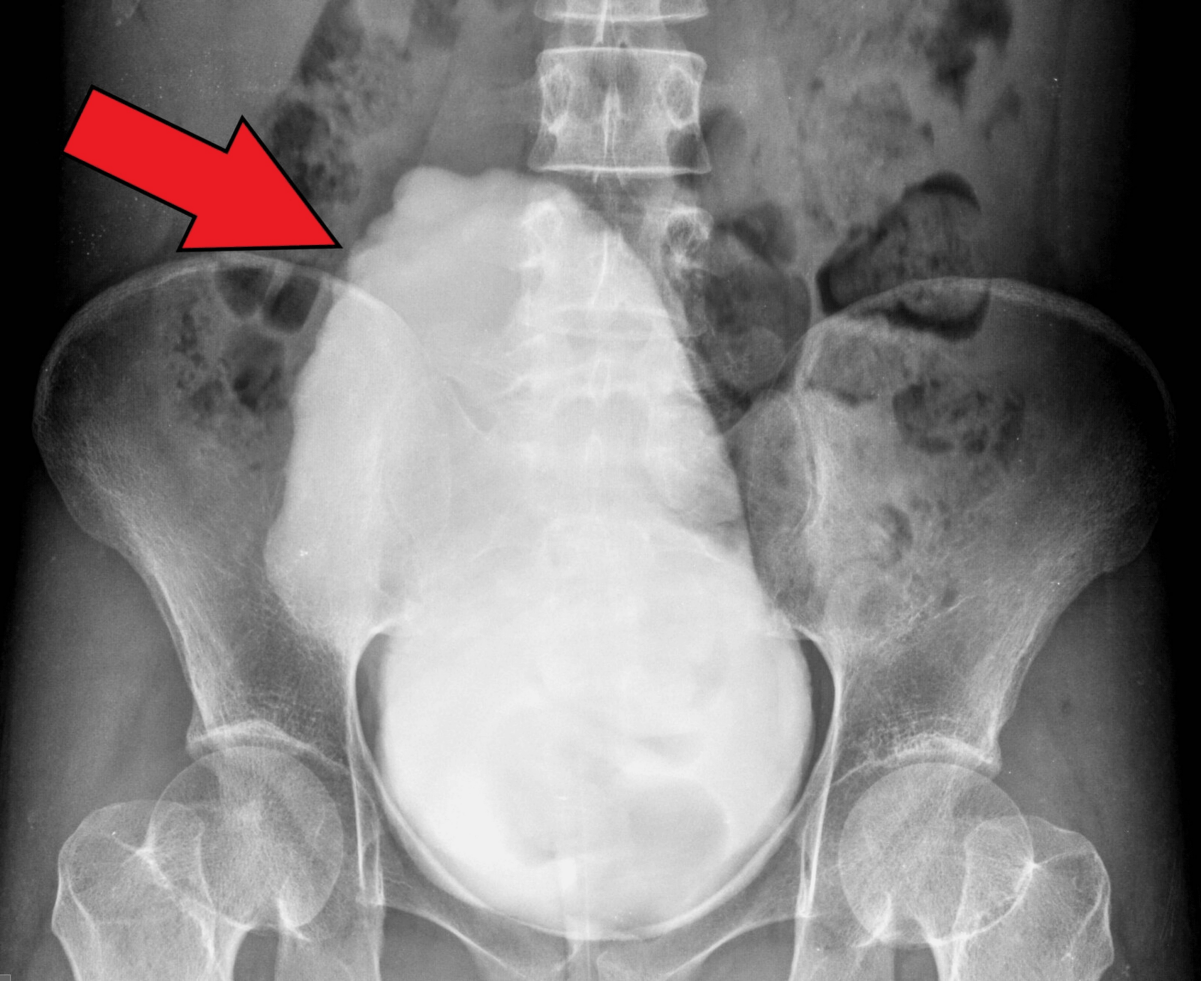
Micturating cystourethrogram showing Christmas tree bladder (red arrow).

A micturating cystourethrogram showed a markedly distended bladder with mucosal irregularities and a cranially facing apex resembling a Christmas tree bladder (Figure 4), indicating a neurogenic bladder.

**Figure 5:**
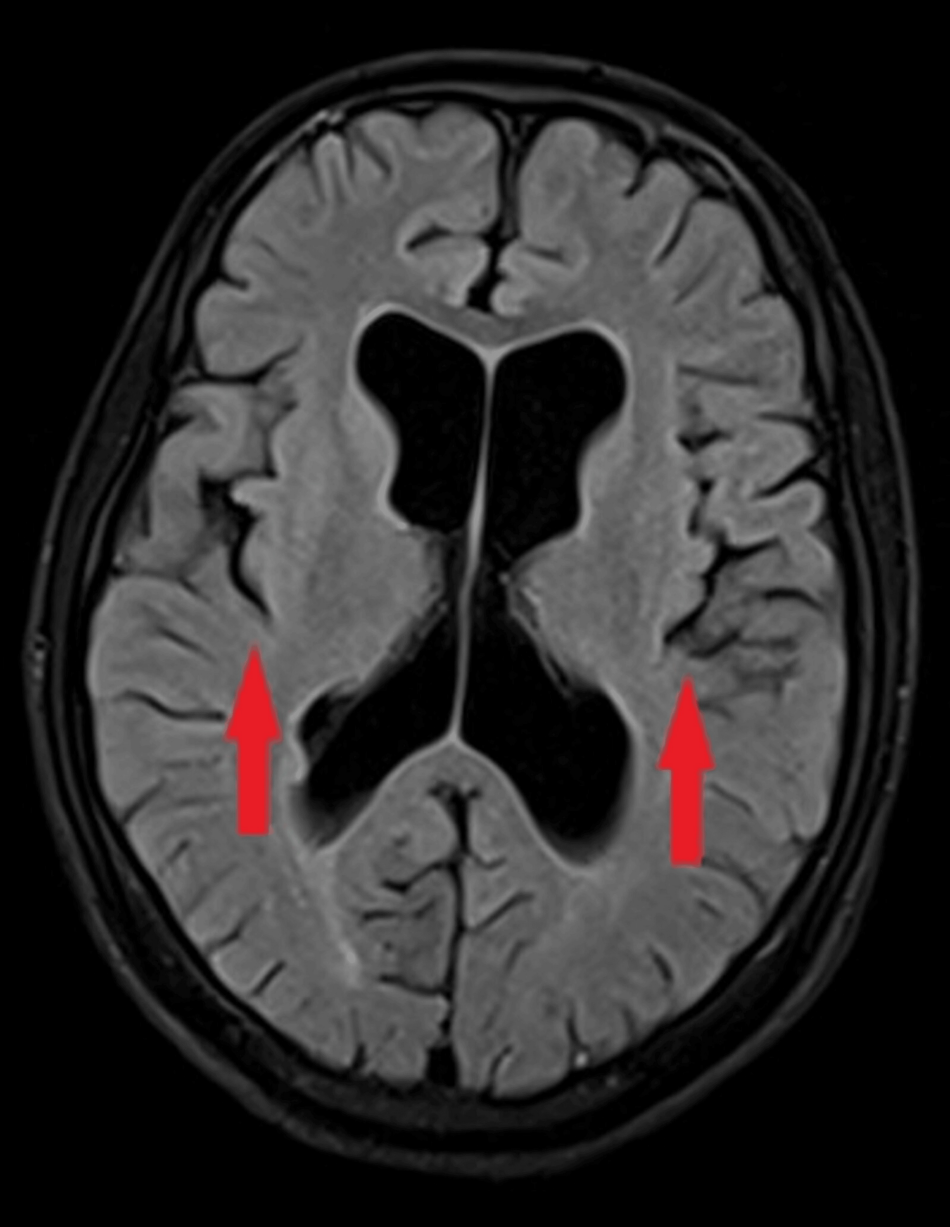
MRI of the brain using T2 FLAIR sequence showing diffuse cerebral atrophy (two red arrows). MRI: Magnetic resonance imaging; FLAIR: Fluid attenuated inversion recovery

**Figure 6:**
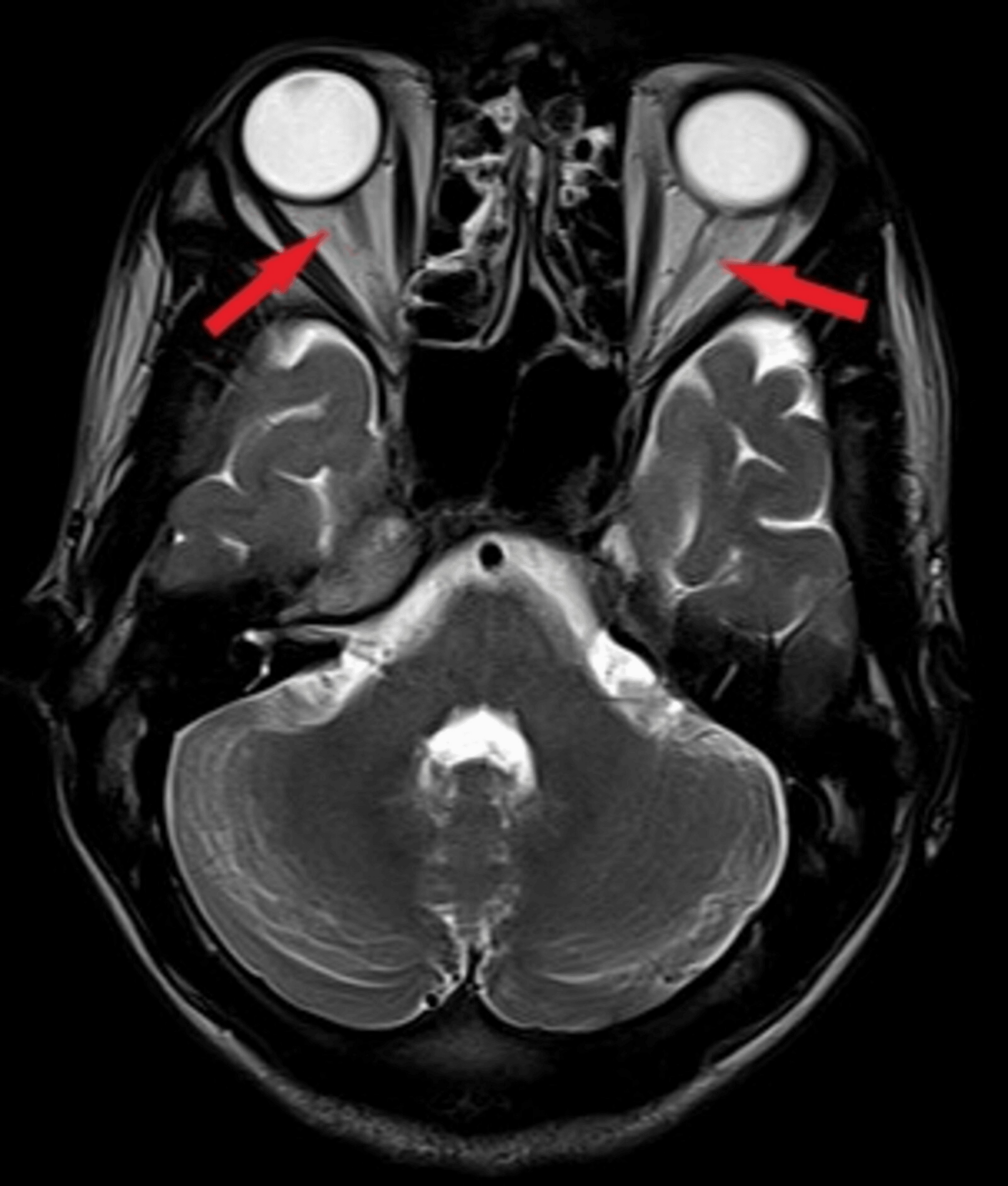
MRI of the brain in T2 axial view showing bilateral optic nerve atrophy (two red arrows). MRI: Magnetic resonance imaging

**Figure 7:**
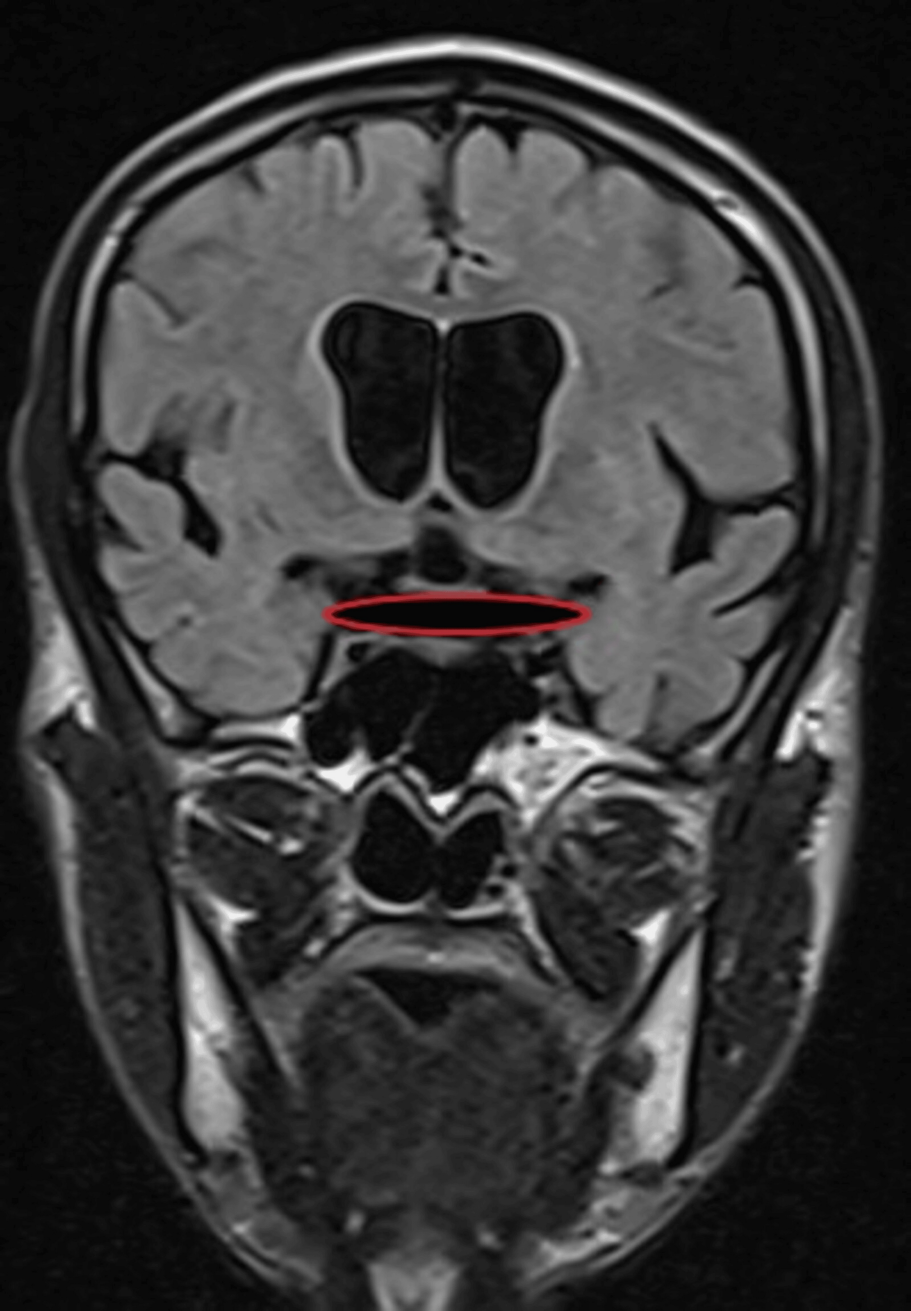
MRI of the brain in T2 FLAIR coronal section showing optic chiasma atrophy (red circle). MRI: Magnetic resonance imaging; FLAIR: Fluid attenuated inversion recovery

Magnetic resonance imaging (MRI) of the brain revealed diffuse cerebral atrophy (Figure 5), along with atrophy of the bilateral optic nerve (Figure 6) and optic chiasma (Figure 7).

**Figure 8:**
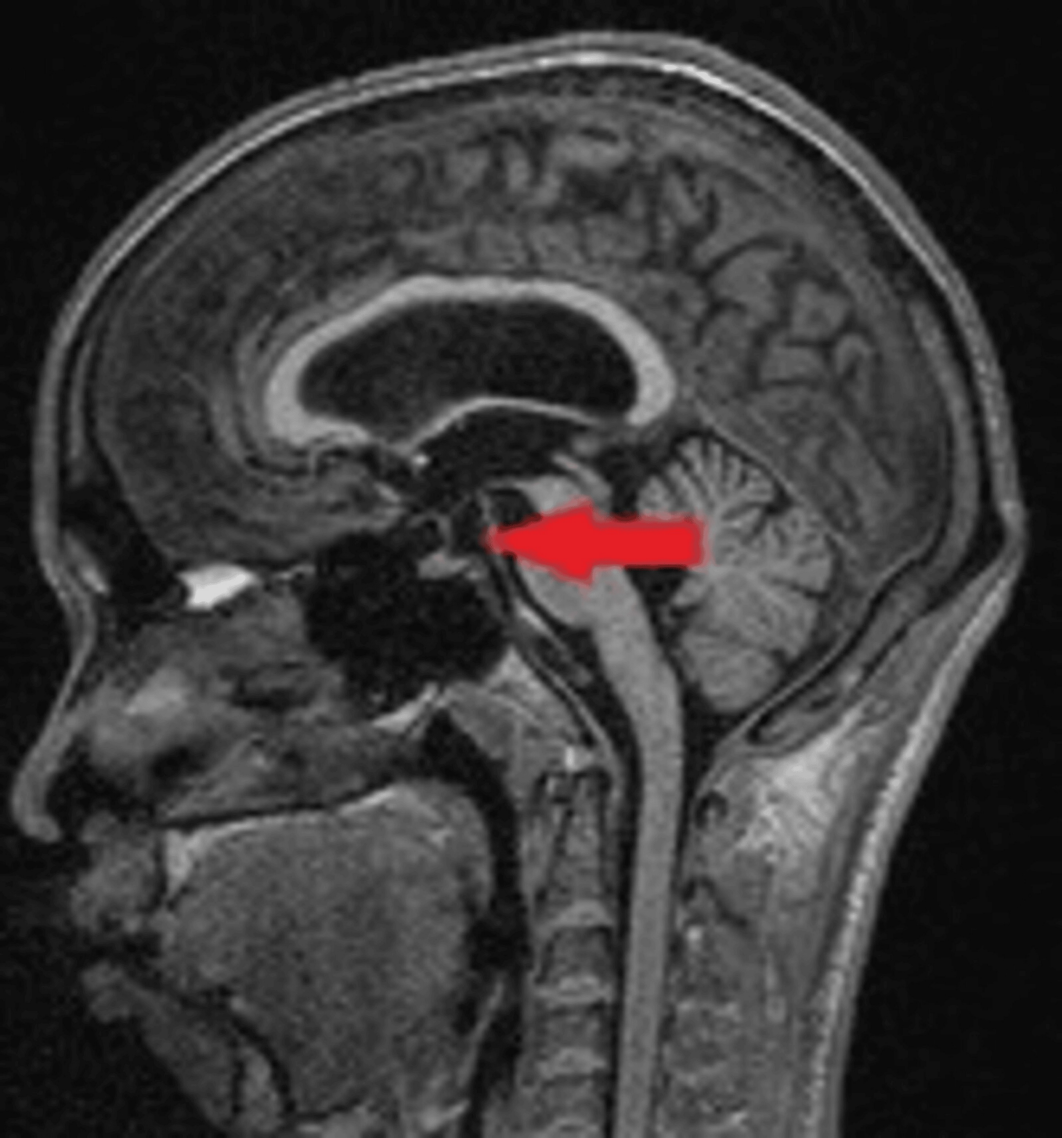
MRI of the brain in T1 MPRAGE sagittal view showing absent posterior pituitary bright spot (red arrow). MRI: Magnetic resonance imaging; MPRAGE: Magnetization prepared rapid gradient echo

The posterior pituitary bright spot was not visualized (Figure 8) in the MRI of the brain.

Low serum arginine vasopressin levels confirmed the diagnosis of central diabetes insipidus.

NX Gen whole exome sequencing genetic testing was conducted due to the suspicion of WFS, and a variant of uncertain significance (VUS) was identified in the WFS-1 gene. The WFS-1 gene's exon 8 exhibited a variant characterized as an insertion-deletion type (indel), NM_006005.3.2142_2165del (p.Asn714_Met722delinsLys), which was located at chromosome 4:6303663. This mutation was associated with WFS-1 (OMIM#222300) and was homozygous and autosomal recessive. The clinical phenotype of the patient overlapped with the manifestation of the condition associated with the WFS-1 gene. In silico prediction tools predict the identified variant to be damaging by MutationTaster. It is absent in the genome aggregation database (gnomAD) and has not been previously reported in the ClinVar database, hence this variant is classified as VUS. The patient was consequently identified with WFS.

The patient had intensive basal-bolus insulin therapy during and after their hospital stay. Oral desmopressin 0.2 mg was initiated, and patient urine output reduced substantially to around 2 L/day. Hearing aids were also prescribed to him after audiological consultation. The patient was trained for self-catheterization of the urinary bladder for neurogenic bladder.

## Discussion

WFS, also known as DIDMOAD syndrome, is indeed an autosomal recessive neurodegenerative disorder characterized by diabetes insipidus, diabetes mellitus, optic atrophy, and deafness. The majority of patients frequently exhibit additional symptoms such as mental retardation, peripheral neuropathy, ataxia, urinary tract atony, and psychiatric issues. The acronym DIDMOAD has been updated to DIDMOADUD to reflect the increased identification of urinary dysfunction [3].

The WFS-1 protein is essential for maintaining neurons and endocrine function. Type 1 diabetes mellitus could be brought on by the destruction of pancreatic beta cells that are highly expressed in the WFS-1 gene [4]. Central diabetes insipidus is believed to be associated with hypothalamic shrinkage and degeneration, in addition to the atrophy of the posterior pituitary gland and the loss of neurons in the supraoptic area that release vasopressin [5]. The loss of function in the vestibulocochlear nuclei and inferior colliculi results in sensorineural deafness.

WFS is categorized into type 1 (WFS-1) or type 2 (WFS-2), distinguished by specific genetic mutations and how symptoms manifest clinically. The sequence of the WFS-1 gene was identified in 1998. The majority of mutations, according to genetic studies, are found in the eighth exon of the WFS-1 gene, which is situated on chromosome 4p16.1. This sequence represents instructions for wolframin, a type of protein that spans the cell membrane and is situated in the endoplasmic reticulum (ER) [6].

The majority of documented genetic alterations consist of missense, nonsense, and splice site changes, along with deletions and insertions [7]. However, a variety of clinical presentations affecting various organs and tissues point to a possible role for mitochondrial deoxyribonucleic acid (DNA). Recessive changes in the CISD2 gene, located at 4q24 (OMIM 604928), have been documented worldwide in individuals with WFS-2. CISD2 encodes a small protein known as CDGSH sulfur domain-containing protein 2, which resides in the intermembrane space of the ER.

Possible differential diagnoses encompass dominant inheritance pattern of optic nerve degeneration, Leber's hereditary optic neuropathy, thiamine-dependent megaloblastic anemia, Mohr-Tranebjaerg syndrome, and mitochondrial illnesses such as maternally transmitted diabetes and deafness [8]. In families with a known causal mutation, prenatal diagnosis is possible, and genetic counseling can be provided to at-risk couples.

WFS's pathophysiology has identified potential targeted therapies aimed at reducing complications. Treatments focus on alleviating ER stress or enhancing mitochondrial function to potentially improve neurological and pancreatic beta cell survival [9,10]. Management primarily addresses symptoms, and the condition often results in premature death due to respiratory failure.

A similar case has been reported in which generic symptoms of WFS are discussed. In the current case report, the significance of early detection and treatment of background urological manifestations in such patients is highlighted [11].

## Conclusions

The symptoms of WFS can be very diverse, hence making an accurate diagnosis is difficult without a multidisciplinary examination and specialist testing to disclose all components of the syndrome. Young individuals with type 1 diabetes mellitus and optic atrophy, jointly, need to be assessed in relation to WFS. To enhance patient prognosis and predict related issues, patients with WFS should be monitored throughout their lifetimes. Collaboration among multiple experts such as endocrinologists, neurologists, ophthalmologists, and urologists is necessary for management. Drug repurposing and gene therapy are two complementary therapeutic approaches being investigated, despite the fact that there are currently no established viable treatments for WFS. Genetic counseling can be given to couples at risk. Recuperation to a regular social life is achievable with appropriate therapy and follow-up.
